# Proteomic analysis of liver tissue from dogs with chronic hepatitis

**DOI:** 10.1371/journal.pone.0208394

**Published:** 2018-11-30

**Authors:** Yuri A. Lawrence, Lawrence J. Dangott, Aline Rodrigues-Hoffmann, Jörg M. Steiner, Jan S. Suchodolski, Jonathan A. Lidbury

**Affiliations:** 1 Gastrointestinal Laboratory, Department of Small Animal Clinical Sciences, College of Veterinary Medicine and Biomedical Sciences, Texas A&M University, College Station, Texas, United States of America; 2 Protein Chemistry Laboratory, Department of Biochemistry and Biophysics, Texas A&M University, College Station, Texas, United States of America; 3 Department of Veterinary Pathobiology, College of Veterinary Medicine and Biomedical Sciences, Texas A&M University, College Station, Texas, United States of America; Centre de Recherche en Cancerologie de Lyon, FRANCE

## Abstract

Chronic hepatitis is the most common hepatic disease in dogs. Copper accumulation is an important cause of chronic hepatitis in dogs; however, the etiology in most dogs cannot be determined. Clinical signs of chronic hepatitis are often non-specific; therefore, this disease is frequently diagnosed in an advanced stage that makes successful intervention less likely. Early diagnosis of chronic hepatitis in dogs would thus be beneficial. The identification of proteins that are differentially expressed in dogs with chronic hepatitis could contribute to the development of novel diagnostic markers for this disease and provide insight into its pathogenesis. The objective of this study was to identify novel proteins that are differentially expressed in the liver of dogs with chronic hepatitis. Hepatic tissue was collected from 8 healthy dogs during ovariohysterectomy and from 8 dogs with histologically confirmed chronic hepatitis. The proteome of the liver samples was extracted by mechanical disruption and detergent-based cell lysis and differentially labeled prior to analysis by 2-dimensional fluorescence difference gel electrophoresis. Spots with an absolute fold change value > 2.0 were selected for further analysis. Protein identification was achieved by nanoflow liquid chromatography tandem mass spectrometry. Differential expression of select proteins was validated by Western blot. Five protein spots were differentially expressed between patients with chronic hepatitis and healthy control dogs. From these 5 protein spots 11 proteins were identified. Differential expression of cytokeratin 18 and annexin 5 were confirmed by Western blot analysis. Differential protein expression was shown between dogs with chronic hepatitis and healthy control dogs. Upregulation of cytokeratin 18 in chronic hepatitis may suggest increased hepatocellular apoptosis and necrosis, whereas upregulation of annexin 5A suggests increased hepatocellular apoptosis. Further studies are needed to determine whether either protein has diagnostic utility.

## Introduction

Chronic hepatitis is the most common type of liver disease in dogs with chronic hepatitis being the most frequently observed form [1]. Chronic hepatitis is associated with apoptosis/necrosis and an inflammatory infiltrate that is often mononuclear. This results in a repair process that can lead to hepatic fibrosis including end-stage liver disease characterized by fibrosis of the liver and the conversion of normal liver architecture into abnormal liver nodules with portal-central vascular anastomoses [2]. End-stage hepatic disease carries a poor long term prognosis with a reported median survival time of 0.4 months [3,4]. Chronic hepatitis, in contrast diagnosed at an earlier stage carries a reported median survival from diagnosis to death of 18.3–36.4 months [3,4]. Cases of chronic hepatitis are often diagnosed at an advanced stage where treatment is less likely to be effective and thus early identification of dogs with chronic hepatitis and hepatic fibrosis, ideally before clinical signs develop, would be beneficial.

The histological evaluation of a liver biopsy specimen continues to be the gold standard for the assessment of hepatocellular necrosis, inflammation, fibrosis, and for a definitive diagnosis of chronic hepatitis. The current lack of robust biomarkers for canine chronic hepatitis restricts the non-invasive evaluation of this disease. A number of non-invasive biomarkers of hepatocellular injury have been identified and validated in dogs and humans. For example, the most widely used biomarker for hepatocellular injury is serum alanine aminotransferase activity [5]. Alanine aminotransferase is predominantly found in the liver with lower level enzyme activities found in skeletal and cardiac muscle. Serum enzyme activity increases are associated with either reversible or irreversible hepatocellular damage [6]. Serum alanine aminotransferase activity has a reported sensitivity for detecting histopathologic evidence of hepatocellular injury in symptomatic dogs that varies from 60% - 76% [7]. Little is known about the sensitivity of biomarkers including alanine aminotransferase to detect hepatobiliary injury in the subclinical disease phase. A recent study by Dirksen et al. found that serum alanine aminotransferase and alkaline phosphatase activities and serum bile acid concentrations have a sensitivity for detecting histologic evidence of copper-associated chronic hepatitis in the subclinical phase of 71%, 35%, and 13% respectively [8]. Additionally, serum ALT activity can also be increased due to extrahepatic diseases such as pancreatitis, enteritis, and endocrinopathies [1]. Therefore, a more sensitive and specific marker of hepatocellular injury in dogs with chronic hepatitis would be beneficial. There is also an unmet need for novel non-invasive diagnostic tests for hepatic fibrosis, hepatic function, and hepatic neoplasia in dogs.

Proteomics is the large-scale characterization of the protein complement of a cell, tissue, or organism and has emerged as a powerful tool in biomarker discovery. Chronic inflammatory hepatobiliary disease has been demonstrated to result in changes to proteins found in hepatic tissues in humans [9]. There is one proteomic study of the canine liver to the authors knowledge. This study evaluated the soluble liver proteome from bull terriers affected with inherited lethal acrodermatitis [10]. Proteomic analysis of canine hepatic tissue from dogs with chronic hepatitis may lead to the identification of novel candidate biomarkers that could later be evaluated in serum or urine as well as having the potential to provide new insights into the pathogenesis of this disease.

The aim of this study was to identify proteins that are differentially expressed between hepatic tissue from dogs with chronic hepatitis and healthy control dogs. We hypothesized that dogs with chronic hepatitis will exhibit differential hepatic protein expression compared to healthy control dogs.

## Materials and methods

### Patients and procedures

Eight canine patients undergoing liver biopsy for routine diagnostic purposes that were histologically confirmed to have chronic hepatitis and 8 healthy control dogs undergoing ovariohysterectomy were enrolled at the Texas A&M University Veterinary Medical Teaching Hospital between September 2014 –August 2017. Informed client consent was obtained for each patient and the study was approved by the Texas A&M University Institutional Animal Care and Use Committee (Animal Use Protocol #2014–320 and 2015–0043). Patients with suspected chronic hepatitis underwent laparoscopic liver biopsy and 5 to 8, 5 mm closed cup forceps liver biopsy specimens were collected. The same 5 mm closed cup forceps were used to collect 3–4 liver biopsy specimens from healthy dogs at the time of ovariohysterectomy. One biopsy specimen, approximately 0.5 g of liver tissue from each patient was flash frozen in liquid nitrogen within 30 minutes of acquisition and stored at -80°C. One liver biopsy specimen was collected in a sterile red top tube for copper quantification by atomic adsorption spectroscopy (expressed as μg/g dry weight) and another was collected and stored in a sterile specimen cup for aerobic and anaerobic culture and susceptibility testing. The remaining liver tissue from dogs in each group was fixed in neutral buffered formalin for routine histological processing. Histological sections from formalin-fixed paraffin embedded tissues were stained with hematoxylin and eosin, picrosirius red, and rhodamine. The diagnosis of chronic hepatitis and healthy control were based on clinical signs, routine serum biochemistry panels, and histological assessment of liver specimens by a board-certified veterinary pathologist according to the World Small Animal Veterinary Association Liver Standardization Group Guidelines[11]. The stage of hepatic fibrosis, the grade of necroinflammatory activity, and the semiquantitative assessment of copper content were assessed using a previously published scoring scheme[2,12].

### 2-Dimensional fluorescence difference gel electrophoresis

#### Protein processing and labeling

Each liver biopsy specimen was placed in a 1.5 mL ground glass pestle and tube tissue grinder and homogenized in 10 mM Tris-HCL/1% CHAPS buffer. The overall protein concentration was determined by Bradford protein assay using bovine serum albumin as a standard. The proteome from each liver sample was precipitated with choloroform/methanol and dissolved in 1 mL of DIGE labeling buffer (30 mM Tris, 7 M urea, 2M Thiourea, 4% CHAPS, pH 8.5 buffer) [13]. Samples were fluorescently labeled by reacting 50 μg of protein with 200 pmol CyDye DIGE Fluors (GE Healthcare). One sample was labeled with Cy3 while the other was labeled with Cy5 to allow for differential labeling. A pooled sample containing equal amounts of each sample was labeled with Cy2. The labeling reactions were quenched with 10 mM lysine.

#### 2- Dimensional difference gel electrophoresis

Cy2-, Cy3- and Cy5-labeled samples were mixed together, and rehydration buffer was added (7 M urea, 2 M thiourea, 4% CHAPS, 0.5% pharmalytes (GE Healthcare), 40 mM dithiothreitol (DTT), and 0.002% bromophenol blue) to a final volume of 450 μl and used to rehydrate an immobilized pH gradient strip (24 cm; pH 3–10NL; GE Healthcare) by passive diffusion at 20°C for 12h. Isoelectric focusing was performed on an IPG Phor 3 horizontal electrophoresis system (GE Healthcare) with a program of 0.5 kV for 1h, holding at 0.5 kV for 5 h, ramping to 1 kV over 1 h, ramping to 8 kV over 3 h, holding at 8 kV until 60 kV*h, and holding at 0.5 kV for 4 h. Each focused strip was then equilibrated in two steps: 15 minutes in a reducing equilibration buffer (6 M urea, 50 mM Tris-HCl, pH 8.8 with 30% (v/v) glycerol, 2% (w/v) SDS, 0.01 bromophenol, and 10 mg/mL DTT), followed by 15 minutes in an alkylating equilibration buffer where DTT was replaced by 25 mg/mL iodoacetamine. The equilibrated IPG strips were then placed directly on top of polymerized 12% SDS gels and sealed with an agarose sealing solution (25 mM Tris, 192 mM, glycine, 0.1% SDS, 0.5% (w/v) agarose, and 0.02% bromophenol blue). Gels were run in cooled tanks with DIGE buffer on an Ettan Dalt-6 (GE Healthcare) at 1 W per gel until the bromophenol blue dye front reached the bottom of the gel.

#### Image acquisition and analysis

Gel images were obtained on a Typhoon FLA 9500 (GE Healthcare) at an excitation wavelength of 473 nm for Cy2, 532 nm for Cy3, and 635 nm for Cy5 labeled samples. The resolution was set at 100 μm. DeCyder 2-D Differential Analysis Software (v 6.5, GE Healthcare) was utilized for data analysis. The software subtracts the background signal using a subtraction algorithm and quantifies spot intensity based on normalized spot volume. The normalization is ratiometric with Cy3 and Cy5 images being expressed as a ratio of the corresponding Cy2 internal standard protein spot. Further, DeCyder was also used to match spots between gels and determine significant changes in protein expression and the absolute fold change ratio. Absolute fold change ratio was derived from the normalized spot volume standardized against the intragel standard providing a measure of the magnitude of expression differences between identified spots. Protein spots present in the gels were matched using Decyder software from which a pick list was generated. Spot detection was verified visually. Statistical significance was determined by a two-sided student's t-test. Post-hoc testing was performed using Bonferroni’s multiple comparisons test and significance was set at q < 0.05. Proteins with an absolute fold change value > 2.0 between dogs with chronic hepatitis and healthy controls were selected for further analysis. All gels were fixed overnight in 10% methanol and 7.5% acetic acid.

#### Spot picking and protein processing

Ettan Spot Handling Workstations (GE Healthcare, Chicago, IL USA) were used to cut out the selected protein spots from a protein gel, perform an in-gel tryptic digestion with recombinant porcine trypsin (Promega, Madison, WI) and extract the peptides from the gel. Extracted trypsin peptides were concentrated by SpeedVac. Protein identification was achieved by nanoflow liquid chromatography tandem mass spectrometry. Subsequently, peptides from the MS were identified using the MASCOT search engine. The MASCOT program (v2.2) searched the mouse genome using the following parameters for protein identification: 1) one missed cleavage by trypsin; 2) monoisotopic peptide masses; 3) peptide mass tolerance of 1.2 Da; and 4) fragment mass tolerance of 0.8 Da. Further, oxidation of methionine (variable modification) and carbamidomethylation (fixed modification) of cysteine were taken into consideration by MASCOT in the protein identification. Peptides were matched to proteins at a minimum of two peptides. Protein identification was verified by Scaffold (Proteome Software, Portland, OR).

#### Western blotting

Select proteins (annexin A5 and cytokeratin 18) that were identified in spots that showed differential expression in hepatic tissue from dogs with chronic hepatitis (based on the 2D-DIGE results) were validated using standard Western blotting techniques. The following antibodies were used: annexin V (1:1000, rabbit polyclonal, Abcam, Cambridge, MA, USA) and cytokeratin 18 (1:20000, mouse monoclonal, Abcam, Cambridge, MA, USA). Liver samples were homogenized in 10 mM Tris-HCL/1% CHAPS buffer. The protein concentration was determined with the Bradford protein assay using a bovine serum albumin standard. Equal protein loading for each lane was confirmed by immunoblotting for anti-Lamin B1 (1:5000, rabbit monoclonal, Abcam, Cambridge, MA, USA). Protein samples were boiled following addition of Laemmli loading dye, separated on precast gels (BIO-RAD 8–16% Mini-PROTEAN TGX), and transferred to polyvinylidene difluoride (PVDF) membranes. PVDF membranes were blocked in 5% milk 0.05% Tween—Tris buffered saline (TTBS) overnight at 4°C, incubated with primary antibodies diluted in 5% milk 0.05% TTBS overnight at 4°C. Membranes were washed in 5% milk 0.05% TTBS and incubated with an HRP-conjugated anti-rabbit or goat IgG 1/20000 dilution in 5% milk 0.05% TTBS. PVDF membranes were washed in 5% milk 0.05% TTBS, incubated in ECL-plus, and the signal was detected using an Amersham Imager 600 (GE Healthcare, Chicago, IL USA). Densitometry was performed using the ImageQuantTM TL (GE Healthcare Life Sciences, Chicago, IL USA) software.

#### Statistical analysis

Differences between groups were analyzed for statistical significance by two-sided student's T-test. Post-hoc testing was performed using Bonferroni’s multiple comparisons test. Significance was set at q < 0.05.

## Results

### Dog demographics and clinical characteristics

The demographics and clinical characteristics for the dogs enrolled into the study are shown in Table 1.

**Table 1:** Demographics and clinical characteristics of dogs enrolled into the study.

Patient	Disease	Age^a^	Sex	Breed	FIS	AS	HVS	CuS	Q Liver Cu	ALT	ALKP	TBILI	GGT
227448	Chronic Hepatitis	12	SF	Labrador Retriever	4	2	1	1	667	466	557	0.2	16
229439	Chronic Hepatitis	6	CM	Doberman Pincher	3	1	0	2	196	989	283	0.3	12
203392	Chronic Hepatitis	9	M	Labrador Retriever	2	2	2	0	463	577	230	0.2	<10
231640	Chronic Hepatitis	5	M	Doberman Pincher	2	2	2	3	3450	750	334	0.2	18
229216	Chronic Hepatitis	4	SF	German Shorthaired	1	2	1	2	1080	231	98	0.4	<10
220269	Chronic Hepatitis	11	SF	Labradoodle	2	2	2	3	1230	663	179	0.3	22
175660	Chronic Hepatitis	14	CM	Rat Terrier	3	0	3	0	170	476	327	<0.1	12
231108	Chronic Hepatitis	9	F	Rottweiler Dog	4	1	0	0	257	121	122	0.2	8
229461	Healthy Control	0.5	F	Catahoula Hog Dog	0	0	2		181				
211715	Healthy Control	6	F	Greyhound Dog	0	0	0		168				
227893	Healthy Control	1	F	Chihuahua	0	0	0		280				
229383	Healthy Control	5	F	Am. Staffordshire Terrier	0	0	2		153				
229057	Healthy Control	1	F	Labrador Retriever	0	0	1		124				
229382	Healthy Control	3	F	Walker Coonhound	0	0	0		96.3				
231847	Healthy Control	0.5	F	Labrador Retriever	0	0	0		316				
229270	Healthy Control	0.5	F	Australian Shepherd	0	0	0		158				

^a^Age (years)

SF–Spayed female, M–Male, CM–Castrated male, F–Intact female, M–Intact male, FIS–Fibrosis score, AS–Activity score, HVS–Hepatocellular vacuolation score, CuS–Copper Score, Q Liver Cu–Quantitative liver copper, ALT–Alanine transaminotransferase, ALKP–Alkaline phosphatase, GGT–Gamma-glutamyl transferase

#### Liver biopsy specimens

All liver biopsies were assessed to be of adequate diagnostic quality with > 12 portal triads per biopsy specimen [14]. The liver biopsy specimens of control dogs were free from any remarkable histopathological changes.

#### Identification of candidate biomarkers for chronic hepatitis

**Fig 1:**
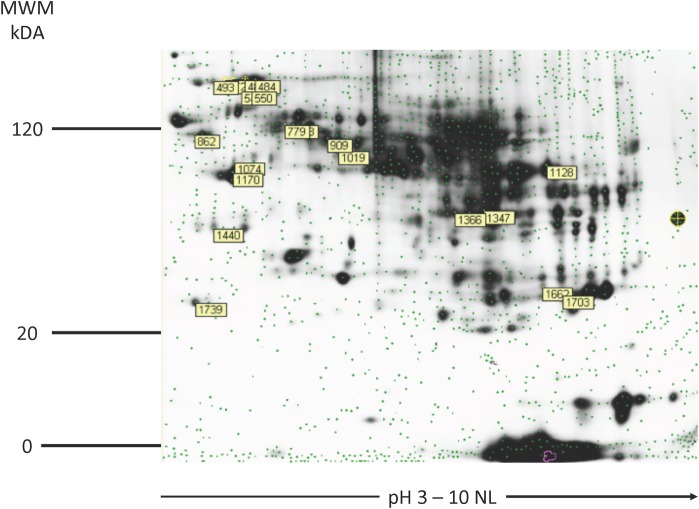
2-D gel image of the master gel with labeled picked proteins spots. Proteome pattern of canine liver tissue by means of 2D –DIGE. For protein analysis, proteins were labeled with Cy2, Cy3, and Cy5, separated in the first dimension using an immobilized pH gradient, and subsequently, in the second dimension, by sodium dodecyl sulfate polyacrylamide gel electrophoresis. Protein spots were detected using a fluorescence scanner (MWM–molecular weight marker; kDA–kilodalton, NL–nonlinear).

Liver tissue specimens from eight dogs with chronic hepatitis and eight healthy control dogs were adequate to show a reproducible liver proteome spot pattern of approximately 2,500 spots. Each spot could contain 1 or multiple proteins. Labeled spots were selected for analysis (Fig 1). Image analysis revealed 5 differentially expressed protein spots (q < 0.05 and fold change > 2.0) that were selected for protein identification.

Altogether 5 differentially expressed protein spots contained 11 different proteins (Table 2). Spot 1440 showed a 3.18-fold increase in abundance (for chronic hepatitis dogs compared to healthy control dogs; q = 0.00095) and contained annexin 5, regucalin, and haptoglobin (Table 1). For protein disulfide–isomerase A4, from spot 472 a 2.33-fold increase was detected (q = 0.0000325). Additionally, we found a 2.24-fold increase in abundance of spot 779 that contained formimidoyltransferase-cyclodeaminase isoform X1, annexin A6, and UDP-glucose 6-dehydrogenase (q = 0.00033) (Table 2).

**Table 2:** Summary of proteins identified from differentially expressed spots.

Spot	Identified Protein	GENE	GO Biological Process	Fold ▽	Peptide No.	q Value	P Value
1047	Succinyl CoA	SUCLG1	cellular metabolic process	2.75	10.00	0.031	0.0062
1047	Keratin, Type II Cytoskeletal 8	KRT8	hepatocyte apoptotic process	2.75	6.00	0.031	0.0062
1047	Keratin, Type I Cytoskeletal 18	KRT18	hepatocyte apoptotic process	2.75	7.00	0.031	0.0062
1440	Annexin A5	ANXA5	negative regulation of apoptotic process	3.18	6.00	0.00095	0.00019
1440	Regucalcin	RGN	L-ascorbic acid biosynthetic process	3.18	8.00	0.00095	0.00019
1440	Haptoglobin	HP	acute—phase response/antioxidant activity	3.18	3.00	0.00095	0.00019
460	Protein Disulfide-Isomerase A4	PDIA4	cell redox homeostasis	2.24	3.00	0.00295	0.00059
460	Gamma-glutamyltransferase 2	TGM3	protein tetramerization	2.24	3.00	0.00295	0.00059
472	Protein Disulfide-Isomerase A4	PDIA4	cell redox homeostasis	2.33	3.00	3.25E-005	6.50E-006
779	Formimidoyltransferase-cyclodeaminase	FTCD	cellular metabolic process	2.24	7.00	3.30E-004	6.60E-005
779	Annexin A6	ANXA6	apoptotic signaling pathway	2.24	6.00	3.30E-004	6.60E-005
779	UDP-glucose 6-dehydrogenase	UDGH	glycosaminoglycan biosynthetic process	2.24	4.00	3.30E-004	6.60E-005

#### Analysis of cytokeratin 18 and annexin 5 in the liver of dogs with chronic hepatitis using immunoblotting

**Fig 2:**
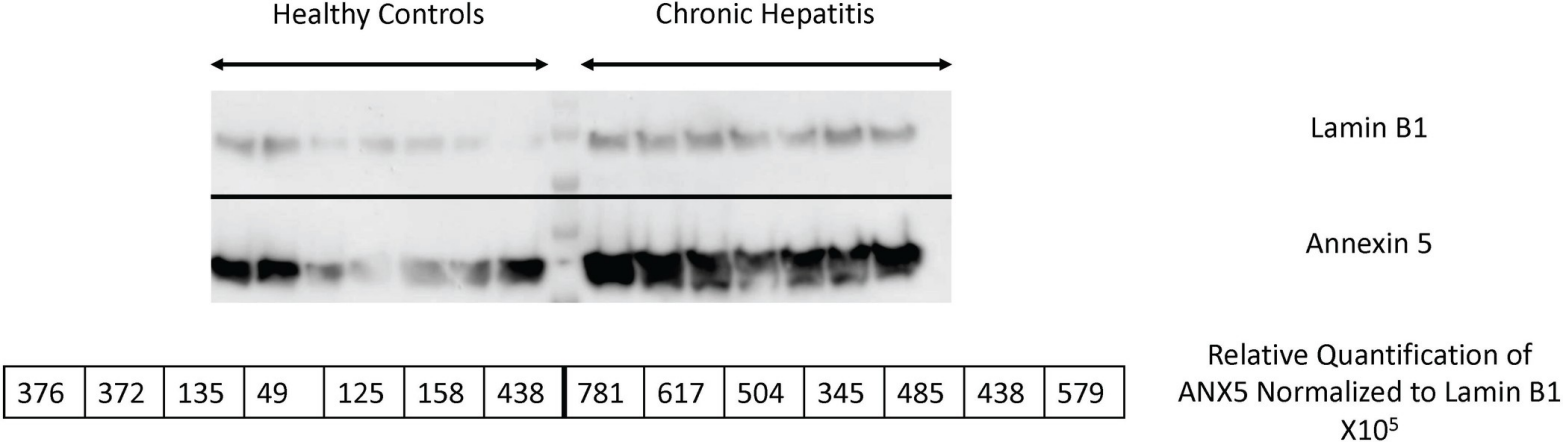
Relative abundance of annexin 5 in the hepatic tissue of dogs with chronic hepatitis by Western blot analysis. Annexin 5 was assessed in the liver proteome of 7 dogs with chronic hepatitis and compared to 7 healthy control dogs. Lamin was used as a loading control.

**Fig 3:**
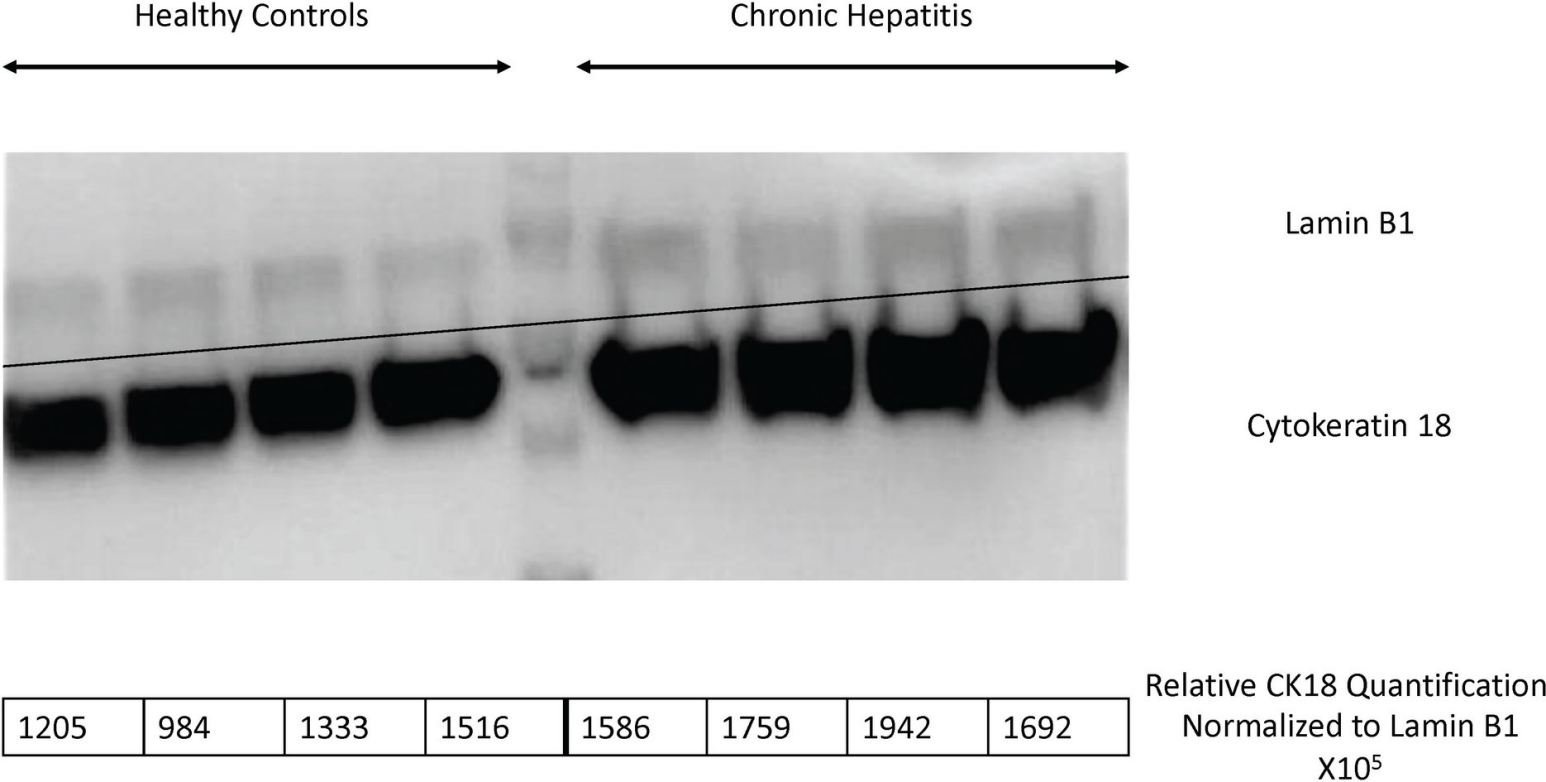
Abundance of cytokeratin 18 in the hepatic tissue of dogs with chronic hepatitis by Western blot analysis. Cytokeratin 18 was assessed in the liver proteome of 4 dogs with chronic hepatitis and compared to 4 healthy control dogs. Lamin was used as a loading control.

Two proteins were selected for Western blot analysis. The proteins, cytokeratin 18 and annexin 5 were confirmed to contribute to the increased relative abundance in the liver proteome of dogs with chronic hepatitis (n = 7, n = 4) compared to healthy controls (n = 7, n = 4), respectively (Table 2; Table 3; Fig 2 and Fig 3). Not all dogs were used to conserve the limited quantity of available protein.

**Table 3:** Patient allocation for Western blot analysis.

Patient ID#	Disease	FIS	Age^a^	Sex	Breed	WB CK18	WB ANX5
227448	Chronic Hepatitis	4	12	SF	Labrador Retriever	X	
229439	Chronic Hepatitis	3	6	CM	Doberman Pincher	X	X
203392	Chronic Hepatitis	2	9	M	Labrador Retriever	X	X
231640	Chronic Hepatitis	2	5	M	Doberman Pincher	X	X
229216	Chronic Hepatitis	1	4	SF	German Shorthaired Pointer		X
220269	Chronic Hepatitis	2	11	SF	Labradoodle		X
175660	Chronic Hepatitis	3	14	CM	Rat Terrier		X
231108	Chronic Hepatitis	4	9	F	Rottweiler Dog		X
229461	Healthy	0	1	F	Catahoula Hog Dog		
211715	Healthy	0	6	F	Greyhound Dog	X	X
227893	Healthy	0	1	F	Chihuahua	X	X
227893	Healthy	0	5	F	Am. Staffordshire Terrier	X	X
229057	Healthy	0	1	F	Labrador Retriever		X
229382	Healthy	0	3	F	Treeing Walker Coonhound	X	X
231847	Healthy	0	1	F	Labrador Retriever		X
229382	Healthy	0	1	F	Australian Shepherd		X

^a^Age (years)

SF–Spayed female, M–Male, CM–Castrated male, F–Intact female, M–Intact male, FIS–Fibrosis score, WB–Western blot, CK18 –Cytokeratin 18, ANX5 –Annexin 5

## Discussion

Our study utilized a gel-based, quantitative proteomics approach to identify differentially expressed proteins in the liver of dogs with chronic hepatitis compared to healthy control dogs. We detected 2,500 protein spots in the liver biopsy samples from dogs. We identified 5 protein spots that showed differential expression with an absolute fold change of at least 200% (2.00 absolute fold change) representing 11 proteins was significantly different between groups. These proteins warrant investigation as potential biomarkers of canine chronic hepatitis. Several of the identified proteins such as cytokeratin 18, cytokeratin 8, annexin 5, and annexin 6 are involved with hepatocyte apoptosis. Only cytokeratin 18 and annexin 5 were validated by Western blot in this study. All other identified proteins need to be validated to confirm these differences.

Hepatocellular apoptosis is a key event in the pathophysiology of many chronic liver diseases of dogs and humans and it is associated with liver fibrogenesis and the development of cirrhosis[15–18]. The effector caspases are activated in the final common pathway of apoptosis. Cytokeratin 18 is the major intermediate filament protein in the liver and the most prominent substrate of caspases during hepatocellular apoptosis[19]. Apoptotic cell death of hepatocytes is associated with release of caspase‐cleaved CK18 fragments in the bloodstream and in contrast, the cytosolic pool of uncleaved CK18 is released from hepatocytes during necrosis[20]. Multiple studies have assessed human serum or plasma CK18 concentrations and suggested the potential use of CK18 fragments as a noninvasive biomarker for the diagnosis or staging of chronic liver disease[21]. Furthermore, several studies of nonalcoholic fatty liver disease in humans have demonstrated that caspase activation and liver cell apoptosis were prominent features of this disease. Additionally, the degree of apoptosis correlated with the severity of hepatitis and the stage of fibrosis[22]. Serum CK18 is a marker of hepatocyte apoptosis in humans and correlates with histologic activity more accurately than serum alanine aminotransferase[23]. Annexin 5A (ANXA5) is a negatively charged phospholipid-binding protein that is widely used as a marker of apoptosis. Annexin 5A binds to negatively charged phospholipids, such as phosphatidylserine that are exposed on the cell surface during apoptosis[24,25]. Increased hepatic annexin 5A expression could therefore indicate ongoing apoptosis. Annexin A5 and CK18 have not been previously evaluated in canine liver disease. The apoptotic pathway may be altered in canine chronic hepatitis and other proteins in this pathway should be further investigated in a targeted study.

This is the first study to evaluate the liver proteome of dogs with chronic hepatitis. However, limitations of our study include the heterogeneity of the dogs in the chronic hepatitis group with one copper-associated chronic hepatitis case and 7 idiopathic chronic hepatitis cases. Examining specific subpopulations of dogs with chronic hepatitis may be advantageous. Another limitation is the fact that we only looked at proteins with an absolute fold change of > 2.0 between groups to avoid false discovery, which eliminated a number of proteins from further analysis. Another limitation is the use of only female dogs as a control group; however, the impact of this limitation is unknown. The healthy control group dogs were significantly younger than the chronic hepatitis dogs due to selection bias. The impact of this limitation is unknown, although, age-related changes in the liver proteome have been described in rats[26]. Finally, this initial study has a limited sample size that could have led to type II error. Further work that includes measurement in blood or urine and validation in a diverse clinical population will be required to establish whether the identified proteins have any diagnostic utility.

Proteomic evaluation of liver tissue from dogs with chronic hepatitis demonstrated differential protein expression compared to healthy controls. The increased expression of two proteins involved in the apoptotic pathway, cytokeratin 18 and annexin 5 was confirmed with Western blotting in these tissues. Further work will be required to establish whether these proteins have diagnostic utility over currently available biomarkers.

## Supporting information

S1 Fig Abundance of annexin 5 in the hepatic tissue of dogs with chronic hepatitis by Western blot analysis. Annexin 5 was assessed in the liver proteome of 7 dogs with chronic hepatitis compared to 7 healthy controls. Lamin was used as a loading control. The unedited image is provided as a supplemental figure.
(TIFF)

S2 Fig Abundance of cytokeratin 18 in the hepatic tissue of dogs with chronic hepatitis by Western blot analysis. Cytokeratin 18 was assessed in the liver proteome of 4 dogs with chronic hepatitis compared to 4 healthy controls. Lamin was used as a loading control. The unedited image is provided as a supplemental figure.
(TIFF)

## References

[1] Poldervaart JH, Favier RP, Penning LC, van den Ingh TSGAM, Rothuizen J. Primary hepatitis in dogs: a retrospective review (2002–2006). J Vet Intern Med. 2009;23: 72–80. 10.1111/j.1939-1676.2008.0215.x 19175724 10.1111/j.1939-1676.2008.0215.x

[2] Cullen JM, van den Ingh T, Bunch SE, Rothuizen J. WSAVA Standards for clinical and histological diagnosis of canine and feline liver disease. 2006.

[3] Sevelius E. and Diagnosis prognosis of chronic hepatitis and cirrhosis in dogs. Journal of Small Animal Practice. 1995;36: 521–528. 10.1111/j.1748-5827.1995.tb02801.x 8926720 10.1111/j.1748-5827.1995.tb02801.x

[4] Raffan E, McCallum A, Scase TJ, Watson PJ. Ascites is a negative prognostic indicator in chronic hepatitis in dogs. J Vet Intern Med. 2009;23: 63–66. 10.1111/j.1939-1676.2008.0230.x 19175722 10.1111/j.1939-1676.2008.0230.x

[5] Dirksen K, Verzijl T, van den Ingh TSGAM, Vernooij JCM, van der Laan LJW, Burgener IA, et al Hepatocyte-derived microRNAs as sensitive serum biomarkers of hepatocellular injury in Labrador retrievers. TVJ. W.B. 2016;211: 75–81. 10.1016/j.tvjl.2016.01.010 27021912 10.1016/j.tvjl.2016.01.010

[6] Lawrence YA, Steiner JM. Laboratory Evaluation of the Liver. Veterinary Clinics: Small Animal Practice. Elsevier; 2017;47: 539–553. 10.1016/j.cvsm.2016.11.005 28063744 10.1016/j.cvsm.2016.11.005

[7] Center SA. Interpretation of Liver Enzymes. Veterinary Clinics: Small Animal Practice. Elsevier; 2007;37: 297–333. 10.1016/j.cvsm.2006.11.009 17336677 10.1016/j.cvsm.2006.11.009

[8] Dirksen K, Burgener IA, Rothuizen J, van den Ingh TSGAM, Penning LC, Spee B, et al Sensitivity and specificity of plasma ALT, ALP, and bile acids for hepatitis in labrador retrievers. J Vet Intern Med. 2017;31: 1017–1027. 10.1111/jvim.14716 28543991 10.1111/jvim.14716 PMC5508325

[9] Mölleken C, Sitek B, Henkel C, Poschmann G, Sipos B, Wiese S, et al Detection of novel biomarkers of liver cirrhosis by proteomic analysis. Hepatology. 2008;49: 1257–1266. 10.1002/hep.22764 19177598 10.1002/hep.22764 PMC2895500

[10] Grider A, Mouat MF, Mauldin EA, Casal ML. Analysis of the liver soluble proteome from bull terriers affected with inherited lethal acrodermatitis. MGM. 2007;92: 249–257. 10.1016/j.ymgme.2007.07.003 17693109 10.1016/j.ymgme.2007.07.003 PMC3345203

[11] Administration FAD. Guidance for industry: bioanalytical method validation US Department of Health and Human Services, Washington, DC 2001.

[12] van den Ingh TSGAM, Rothuizen J, Cupery R. Chronic active hepatitis with cirrhosis in the Doberman Pinscher. Veterinary Quarterly. 2011;10: 84–89. 10.1080/01652176.1988.9694154 3413974 10.1080/01652176.1988.9694154

[13] Wessel D, Flügge UI. A method for the quantitative recovery of protein in dilute solution in the presence of detergents and lipids. Analytical Biochemistry. 1984;138: 141–143. 10.1016/0003-2697(84)90782-6 6731838 10.1016/0003-2697(84)90782-6

[14] Kemp SD, Zimmerman KL, Panciera DL, Monroe WE, Leib MS, Lanz OI. A comparison of liver sampling techniques in dogs. J Vet Intern Med. 2015;29: 51–57. 10.1111/jvim.12508 25417960 10.1111/jvim.12508 PMC4858056

[15] Ribeiro PS, Cortez-Pinto H, Solá S, Castro RE, Ramalho RM, Baptista A, et al Hepatocyte apoptosis, expression of death receptors, and activation of NF-κB in the liver of nonalcoholic and alcoholic steatohepatitis patients. The American Journal of Gastroenterology 2004;99: 1708–1717. 10.1111/j.1572-0241.2004.40009.x 15330907 10.1111/j.1572-0241.2004.40009.x

[16] Bexfield NH, Andres-Abdo C, Scase TJ, Constantino-Casas F, Watson PJ. Chronic hepatitis in the English springer spaniel: clinical presentation, histological description and outcome. Veterinary Record. 2011;169: 415–415. 10.1136/vr.d4665 21852307 10.1136/vr.d4665 PMC3361955

[17] Thornburg LP. Histomorphological and immunohistochemical studies of chronic active hepatitis in doberman pinschers. Vet Pathol. 2016;35: 380–385. 10.1177/030098589803500507 9754543 10.1177/030098589803500507

[18] Bantel H, Schulze-Osthoff K. Apoptosis in hepatitis C virus infection. Cell Death and Differentiation 2003 10.1038/sj.cdd.4401119 12655346 10.1038/sj.cdd.4401119

[19] Linder S, Havelka AM, Ueno T, Shoshan MC. Determining tumor apoptosis and necrosis in patient serum using cytokeratin 18 as a biomarker. Cancer Letters. 2004;214: 1–9. 10.1016/j.canlet.2004.06.032 15331168 10.1016/j.canlet.2004.06.032

[20] Leers MPG, Kölgen W, Björklund V, Bergman T, Tribbick G, Persson B, et al Immunocytochemical detection and mapping of a cytokeratin 18 neo‐epitope exposed during early apoptosis. The Journal of Pathology. 1999;187: 567–572. 10.1002/(SICI)1096-9896(199904)187:5<567::AID-PATH288>3.0.CO;2-J 10398123 10.1002/(SICI)1096-9896(199904)187:5<567::AID-PATH288>3.0.CO;2-J

[21] YILMAZ Y. Systematic review: caspase‐cleaved fragments of cytokeratin 18 –the promises and challenges of a biomarker for chronic liver disease. Alimentary Pharmacology & Therapeutics. 2009;30: 1103–1109. 10.1111/j.1365-2036.2009.04148.x 19769633 10.1111/j.1365-2036.2009.04148.x

[22] Lebensztejn DM, Wierzbicka A, Socha P, Pronicki M, Skiba E, Werpachowska I, et al Cytokeratin-18 and hyaluronic acid levels predict liver fibrosis in children with non-alcoholic fatty liver disease. Acta Biochimica Polonica. 2011;58. 22140659

[23] Tsutsui M, Tanaka N, Kawakubo M, Sheena Y, Horiuchi A, Komatsu M, et al Serum fragmented cytokeratin 18 levels reflect the histologic activity score of nonalcoholic fatty liver disease more accurately than serum alanine aminotransferase levels. Journal of Clinical Gastroenterology. 2010;44: 440 10.1097/MCG.0b013e3181bdefe2 20104187 10.1097/MCG.0b013e3181bdefe2

[24] Hawkins TE, Das D, Young B, Moss SE. DT40 cells lacking the Ca2+-binding protein annexin 5 are resistant to Ca2+-dependent apoptosis. PNAS. 2002;99: 8054–8059. 10.1073/pnas.132598099 12060752 10.1073/pnas.132598099 PMC123019

[25] Hayes MJ, Moss SE. Annexins and disease. Biochemical and Biophysical Research Communications. 2004;322: 1166–1170. 10.1016/j.bbrc.2004.07.124 15336964 10.1016/j.bbrc.2004.07.124

[26] Ori A, Toyama BH, Harris MS, Bock T, Iskar M, Bork P, et al Integrated transcriptome and proteome analyses reveal organ-specific proteome deterioration in old rats. Cell Systems. 2015;1: 224–237. 10.1016/j.cels.2015.08.012 27135913 10.1016/j.cels.2015.08.012 PMC4802414

