# Supplementary figures and images for: Proteomic analysis of liver tissue from dogs with chronic hepatitis
Source: PLoS One. 2018 Nov 30;13(11):e0208394. doi: 10.1371/journal.pone.0208394 (PMC6267964; doi:10.1371/journal.pone.0208394)

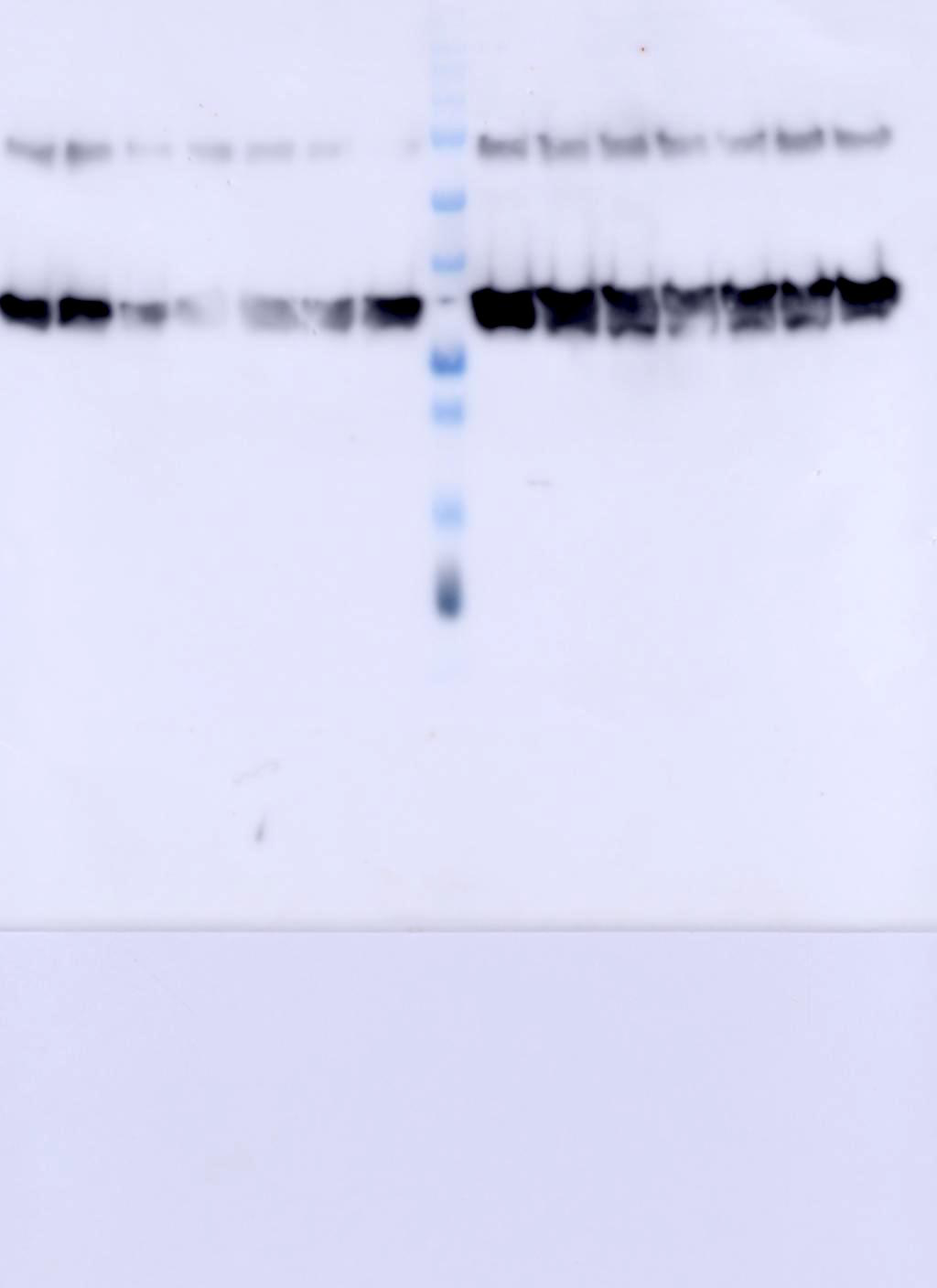

Supplement: S1 Fig — Annexin 5 was assessed in the liver proteome of 7 dogs with chronic hepatitis compared to 7 healthy controls. Lamin was used as a loading control. The unedited image is provided as a supplemental figure. (TIFF) [file pone.0208394.s001.tiff]

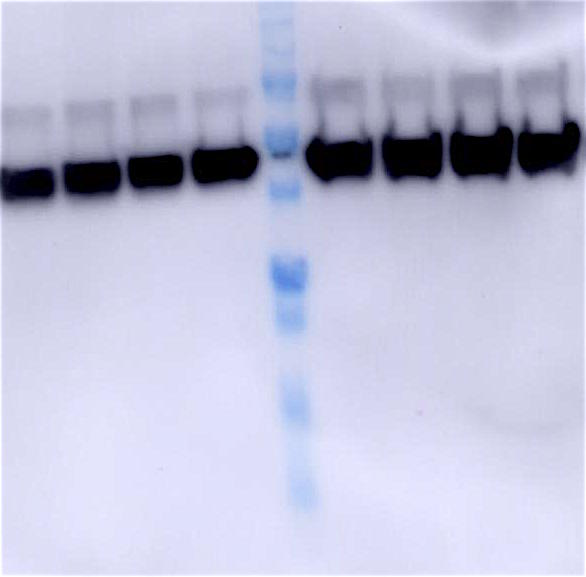

Supplement: S2 Fig — Cytokeratin 18 was assessed in the liver proteome of 4 dogs with chronic hepatitis compared to 4 healthy controls. Lamin was used as a loading control. The unedited image is provided as a supplemental figure. (TIFF) [file pone.0208394.s002.tiff]
